# The Muriate of Cocaine in Opthalmic Surgery

**Published:** 1884-12

**Authors:** 


THE MURIATE OF COCAINE IN OPTHALMIC SURGERY. By C. J. Sundy, A. M., M. D.—The antonishing results of this new local anæsthetic in its effects upon delicate tissues, such as the conjunctive of the eye, in operations upon that organ, are clearly set forth, and its value demonstrated. The effect of this obtunder of sensibility is described, and its value appreciated by the author of this pamphlet which is a reprint from the *Physicians* and *Surgeon.*

